# A systematic review of knowledge, attitude and practice of pharmacogenomics in pediatric oncology patients

**DOI:** 10.1002/prp2.1150

**Published:** 2023-11-27

**Authors:** Claire Moore, Smaro Lazaraki, Tayla Stenta, Marliese Alexander, Rachel Phan Nguyen, David A. Elliott, Rachel Conyers

**Affiliations:** ^1^ Pharmacogenomics Team Murdoch Children's Research Institute Parkville Victoria Australia; ^2^ Department of Paediatrics The University of Melbourne Parkville Victoria Australia; ^3^ Health Sciences Library Royal Melbourne Hospital, Melbourne Health Parkville Victoria Australia; ^4^ Sir Peter MacCallum Department of Oncology The University of Melbourne Parkville Victoria Australia; ^5^ Pharmacy Department Peter MacCallum Cancer Centre Melbourne Victoria Australia; ^6^ St Vincent’s Hospital Fitzroy Victoria Australia; ^7^ The Novo Nordisk Foundation Centre for Stem Cell Medicine, ReNEW, Melbourne Node Parkville Victoria Australia; ^8^ Children's Cancer Centre, The Royal Children's Hospital Parkville Victoria Australia

**Keywords:** Australia, consumer, healthcare professional, oncology, pharmacogenomics, survey

## Abstract

Pharmacogenomics remains underutilized in clinical practice, despite the existence of internationally recognized, evidence‐based guidelines. This systematic review aims to understand enablers and barriers to pharmacogenomics implementation in pediatric oncology by assessing the knowledge, attitudes, and practice of healthcare professionals and consumers. Medline, Embase, Emcare, and PsycINFO database searches identified 146 relevant studies of which only three met the inclusion criteria. These studies reveal that consumers were concerned with pharmacogenomic test costs, insurance discrimination, data sharing, and privacy. Healthcare professionals possessed mostly positive attitudes toward pharmacogenomic testing yet identified lack of experience and training as barriers to implementation. Education emerged as the key enabler, reported in all three studies and both healthcare professionals and consumer groups. However, despite the need for education, no studies utilizing a pediatric oncology consumer or healthcare professional group have reported on the implementation or analysis of a pharmacogenomic education program in pediatric oncology. Increased access to guidelines, expert collaborations and additional guidance interpreting results were further enablers established by healthcare professionals. The themes identified mirror those reported in broader pediatric genetic testing literature. As only a small number of studies met inclusion criteria for this review, further research is warranted to elicit implementation determinants and advance pediatric pharmacogenomics.

**Abbreviations**
ADR: adverse drug reaction
AYA: adolescent and young adult
CPIC: clinical pharmacogenetics implementation consortium
DPWG: Dutch pharmacogenetic working group
EMR: electronic medical record
G6PD: Glucose‐6‐phosphate dehydrogenase
KAP: knowledge, attitudes, and practice
MMAT: mixed method appraisal tool
NUDT15: Nudix hydrolase 15
PGx: pharmacogenomics
PRISMA: preferred reporting items for systematic reviews and meta‐analyses
TPMT: thiopurine methyltransferase

## 1 INTRODUCTION

Pharmacogenomics is a branch of personalized medicine where an individual's drug therapy can be tailored according to their genome.^1^ As genes influence the way drugs act in the body (pharmacokinetics and pharmacodynamics), genetic variations influence drug efficacy and toxicity between individuals. Adverse drug reactions (ADRs) that occur with medicine use are a serious health concern and place a significant burden on healthcare systems.^2^ A 2022 study showed that ADR‐linked hospitalizations account for 16.5% of total admissions in the U.K. with an associated mortality rate of 0.34%.^2^ Similarly, up to 20% of hospital admissions have been attributed to ADRs in some Australian settings, most of which are preventable.^3^

The conceptual framework underpinning pharmacogenomics (or pharmacogenetics) was established in the 1950s,^1^ although clear guidelines for optimizing medications based on pharmacogenomic results were not developed until decades later.^4^ In response to a recognized need to support pharmacogenomic integration into clinical care, two international consortia, the Clinical Pharmacogenetics Implementation Consortium (CPIC)^5^ and the Dutch Pharmacogenetic Working Group (DPWG)^6^ were formed in 2005 and 2009, respectively. These international groups have developed, curated, and published evidence‐based pharmacogenomic guidelines that are freely available and routinely updated. However, despite this, the implementation of pharmacogenomics into clinical practice has remained slow, particularly in the pediatric setting.^1^

Currently, the most well‐studied pharmacogene with personalized prescribing in pediatric childhood cancer therapy is thiopurine methyltransferase (TPMT). The TPMT enzyme metabolizes mercaptopurine (6‐MP), which is a thiopurine drug used as maintenance therapy for patients with acute lymphoblastic leukemia. 6‐MP is taken orally daily with dose adjustments based on myelosuppression, its main toxicity.^7^ TPMT genes (and subsequent alleles) can be defined as high, low, or normal functioning. Individuals with one known low‐functioning allele are considered intermediate metabolizers, while patients with two low‐functioning alleles are poor metabolizers. The remaining patients are considered normal metabolizers. International CPIC and DPWG recommendations for 6‐MP prescribing include starting dose reductions according to TPMT phenotype.^8^ This dosing approach has reduced the risk of life‐threatening myelosuppression without compromising relapse rate.^8^

Pediatric patients with oncology stand to benefit significantly from pharmacogenomics implementation across cancer and supportive care therapies, given the high incidence of medicine‐related adverse events in this group and their sustained polypharmacy.^9^ Up to 60% of pediatric patients with cancer will experience an ADR during their treatment.^9^ A recent landmark adult study of almost 7000 European patients proved pre‐emptive pharmacogenomic‐informed medication dosing significantly reduced ADRs.^10^ Pediatric patients may also benefit from pre‐emptive testing, although ontology, co‐morbidities, number and types of concomitant medications, and diseases may influence this. As such, a gap in the current literature exists.

To understand why pharmacogenomics remains underutilized, barriers to implementation must be identified. Beyond pediatric oncology, reported barriers and enablers to implementation of pharmacogenomics in adult cohorts include lack of education; accessibility, and appropriate funding models.^11^ This systematic review aims to report on knowledge, attitudes, and practice (KAP) studies for pharmacogenomics in pediatric patients with oncology, from both a consumer and healthcare professional perspective to better describe the barriers and enablers to implementation. In addition, educational approaches to pharmacogenomics programs will be described.

## 2 METHODS

This review was conducted according to the guidelines for Preferred Reporting Items for Systematic Reviews and Meta‐Analysis Statement (PRISMA)^12^ and registered with the International Prospective Register of Systematic Reviews (PROSPERO)^13^ in accordance with PRISMA‐P^14^ guidelines.

### 2.1 Eligibility criteria

Randomized, non‐randomized, and cohort studies met inclusion criteria. Studies had to involve KAP, or education related to pharmacogenomics for pediatric or adolescent patients aged <18 years. In addition, patients had to have had a cancer diagnosis or be hematopoietic stem cell recipients. Articles had to be available in full text and be published in English no earlier than 2012. Research of KAP or education could involve studying healthcare professionals or patients/caregivers or both.

### 2.2 Search methods

Advanced literature searches were conducted in November 2022 in the following electronic databases on the Ovid platform: Medline, Embase, Emcare, and PsycINFO.

Articles were limited to the last 10 years and English only. In Medline, the search strategy consisted of a combination of exploded subject headings (MESH) and various keywords to identify the literature. Subject headings applied in Medline included: “Pharmacogenetics”, “Neoplasms”, “Stem cell transplantation”, “Practice patterns, “Physicians”, “child”. The subjects were combined in their associated cluster groups with keywords where all word variations were searched. Keywords included: “pgx”, “cancer”, “clinicians' willingness”.

The adjacency operator was applied in some instances that linked words in proximity to one another. To further refine the search, the pediatrics filter was used. The “AND” operator was then applied to combine all separate concepts and yield relevant results. Searches in Embase, Emcare, and PsycINFO followed a similar strategy to the Medline search with variations according to each database's own subject thesaurus. A detailed search strategy will be provided in Appendix S1.

### 2.3 Article appraisal

Results were uploaded to the systematic review software Covidence.^15^ Covidence is a web‐based collaboration software platform that streamlines the production of systematic and other literature reviews.^15^ Any duplicates were removed. Three reviewers independently reviewed abstracts and then full text for inclusion and exclusion criteria in a two‐stage screening process. Any conflicts were resolved through collaborative discussion leading to consensus. Two reviewers were involved in data extraction. Any one of the reviewers extracted data using standardized pre‐defined tables in Covidence in the fields of study particulars, participant demographics, aims and outcomes, incorporation of pharmacogenomic testing, educational approach, and evaluation used. A second reviewer independently checked the entered data.

### 2.4 Quality assessment of included studies

Each study was assessed for risk of bias by a reviewer using the Mixed Method Appraisal Tool (MMAT).^16^ The MMAT is a validated tool used to appraise qualitative, quantitative, and mixed methods studies included in systematic reviews.^16^ Methodological quality criteria for quantitative descriptive studies assess the clarity of the research question, whether data allow the research question to be addressed, the relevance of the sampling strategy, whether the sample is representative of the target population, whether measurements are appropriate, the risk of nonresponse bias, and appropriateness of statistical analysis.^16^ Each quality assessment was independently checked by a second reviewer, and conflicts were resolved through discussion.

### 2.5 Data analysis

The primary study outcomes of interest were KAP descriptions and the educational approach in patients and their caregivers or healthcare professionals with respect to pharmacogenomic testing in pediatric oncology. Data were analyzed for common themes reported by the studies. Data were analyzed in two subgroups, patients/caregivers and healthcare professionals, due to anticipated differences in both KAP and educational needs/approach.

## 3 RESULTS

### 3.1 Study selection

**FIGURE 1:**
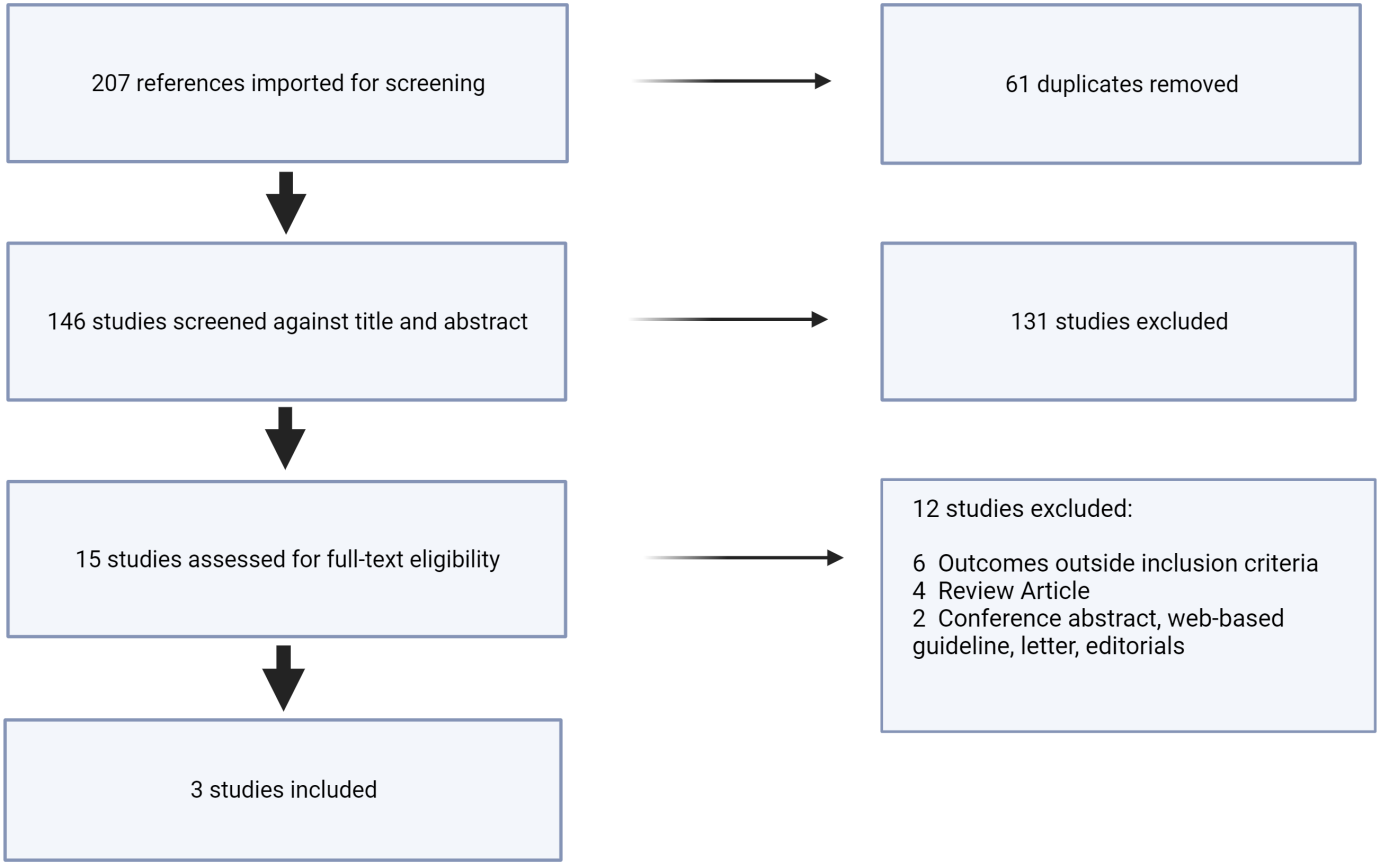
PRISMA flow chart. Created with BioRender.com.

Following a systematic search strategy, 207 articles were identified. With 61 duplicates identified a final 146 studies underwent title and abstract screening. This was performed by three blinded experts. Secondary selection involved the review of 15 full texts (131 studies excluded) to establish article relevance. Following three experts' full‐text screening, three studies fulfilled the inclusion criteria. Figure 1 shows the PRISMA flow chart of the study selection process.

### 3.2 Study characteristics

A descriptive study surveyed 368 oncology nurses in North Carolina, USA, in 2014 to report on knowledge and attitudes with respect to pharmacogenomic testing.^17^ Oncology nurses were identified through the North Carolina Board of Nursing and were invited to participate in the survey. At least one of the nurses had formal pediatric oncology training. The surveys included demographic, genomic, and pharmacogenomic questions, and regression models were generated to identify indicator variables for pharmacogenomic knowledge and attitudes.^17^

The second United States–based study reported on KAP outcomes in both pediatric oncology healthcare professionals and patient caregivers.^18^ Participants were identified through a freestanding academic pediatric hospital. In total, 29 healthcare professionals and 114 patient caregivers completed surveys in 2017 to ascertain their knowledge of and comfort with pharmacogenomic testing. The healthcare professionals were assessed prior to the initiation of a comprehensive hospital‐wide pharmacogenomic program and 15 months later with the study ongoing. Caregiver responses were analyzed in two groups (i) those who chose to participate in the larger pharmacogenomic study and (ii) those who refused.

The third study was a descriptive cross‐sectional study, where surveys were administered to 184 physicians and pharmacists in the Children's Cancer Hospital Egypt 57 357 in 2017.^19^ The surveys collected demographic information and assessed healthcare professionals' knowledge of and attitudes toward pharmacogenomic testing. Provider perspectives on pharmacogenetic testing were also elicited, particularly focused on the anticipated challenges of clinical implementation.

The systematic review of the three studies comprised data extraction across a number of parameters including study characteristics and KAP or education attributes. Table 1 describes the characteristics of the included studies.

**TABLE 1:** Characteristics of included studies.

Population	Study specifics	Participants (*n*)	Study design	Study outcomes
Healthcare professionals	Dodson,^17^ 2014, USA	368	Survey	KAP
	Nagy,^19^ 2020, Egypt	184	Survey	KAP
Mixed	Mowbray,^18^ 2022, USA	29 HCP 114 caregivers	Survey	KAP

### 3.3 Quality assessment of included studies

Studies were compared against the MMAT seven methodological quality criteria. Dodson had a low‐survey response rate but obtained an adequate sample size to demonstrate sufficient power—fulfilling all seven criteria. There was a lack of detail surrounding how suitable participants were identified, and what proportion of eligible participants were approached for inclusion in both Mowbray and Nagy's studies. In addition, both these studies had low‐sample sizes from a single center, meaning six criteria were fulfilled. Overall, the three articles were deemed to be of good quality. The quality assessment is shown in Table 2.

**TABLE 2:** Quality assessment of included studies using the mixed methods appraisal tool.^16^

**Methodological quality criteria** **S1. Are there clear research questions?**			
	Dodson	Mowbray	Nagy
Result	✓	✓	✓
**Methodological quality criteria** **S2. Do the collected data allow to address the research question?**			
	Dodson	Mowbray	Nagy
Result	✓	✓	✓
**Methodological quality criteria** **1. Is the sampling strategy relevant to address the research question?**			
	Dodson	Mowbray	Nagy
Result	✓	✓*	✓*
Comments	* Lacking detail on how suitable healthcare professionals were identified and what proportion of eligible participants were approached for inclusion.		
**Methodological quality criteria** **2. Is the sample representative of the target population?**			
	Dodson	Mowbray	Nagy
Result	✓	?^#^	?^$^
Comments	# Small sample size for healthcare professionals, single‐center study.		
	$ Single‐center study with small sample size.		
**Methodological quality criteria** **3. Are the measurements appropriate?**			
	Dodson	Mowbray	Nagy
Result	✓	✓	✓
**Methodological quality criteria** **4. Is the risk of nonresponse bias low?**			
	Dodson	Mowbray	Nagy
Result	✓	✓^%^	✓
Comments	% Low response rate, however sample size allows for sufficient power.		
**Methodological quality criteria** **5. Is the statistical analysis appropriate to answer the research question?**			
	Dodson	Mowbray	Nagy
Result	✓	✓	✓
Was the study good quality overall?			
	Dodson	Mowbray	Nagy
Result	✓	✓	✓

### 3.4 KAP of consumers

**FIGURE 2:**
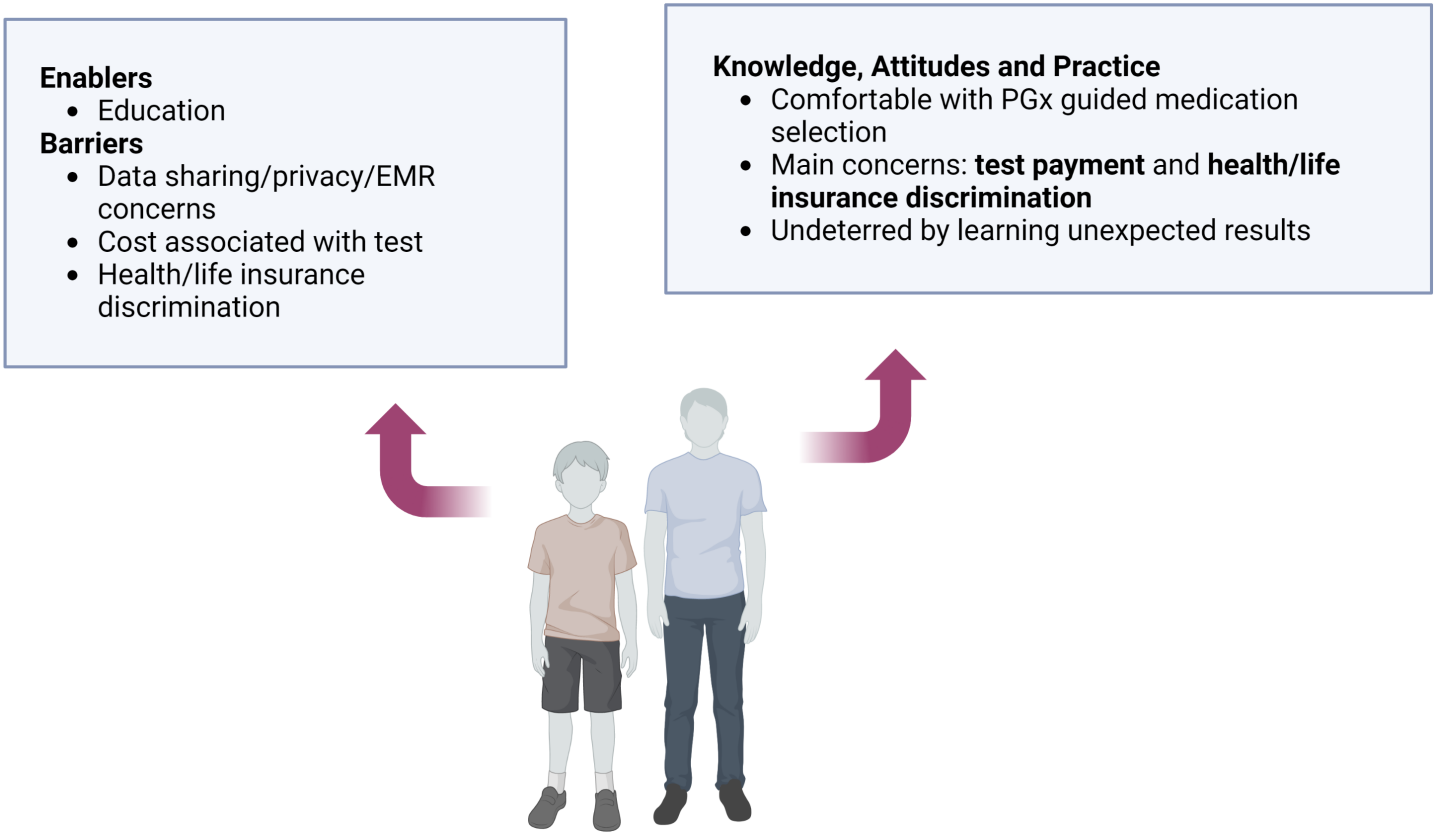
Key themes and attributes identified within consumer surveys.^18^ Created with BioRender.com. EMR, electronic medical record; PGx, pharmacogenomics.

KAP related to pharmacogenomics from a consumer perspective arising from this review are summarized in Table 3 with barrier and enabler themes annotated in Figure 2. Only one study reported on KAP outcomes from a consumer perspective.^18^ This study surveyed caregivers of pediatric oncology patients and identified a lack of experience with medication‐related genetic testing. Only one respondent was confident they had been tested to see how they might respond to a drug. However, the sub‐group that consented to the larger pharmacogenomics study had a better understanding of the role of genetic testing in healthcare in general. Respondents in both groups were most concerned about payment associated with pharmacogenomic tests and possible health or life insurance discrimination. There were also concerns regarding data sharing beyond the treating oncologist and the incorporation of results into electronic medical records. There were no studies that reported on KAP outcomes from a patient perspective.

**TABLE 3:** Attributes of consumer knowledge, attitudes and practice related to pharmacogenomics in pediatric oncology of included studies.^18^

	Attribute
Knowledge	1. There was much better understanding of genetic testing in healthcare among respondents that had agreed to participate in a pharmacogenomic study than those that refused
Attitudes	1. Respondents were most comfortable with pharmacogenomic testing to help identify medication selection
	2. Some discomfort with sharing genetic information beyond treating oncologist, or in electronic medical record
	3. Concerns regarding health or life insurance discrimination
	4. Concerns regarding consumer test costs
	5. Most respondents were not deterred by learning unexpected results
Practice	1. Only one respondent (of 114) knew they had been tested to see how they might respond to a drug

### 3.5 KAP of healthcare professionals

**FIGURE 3:**
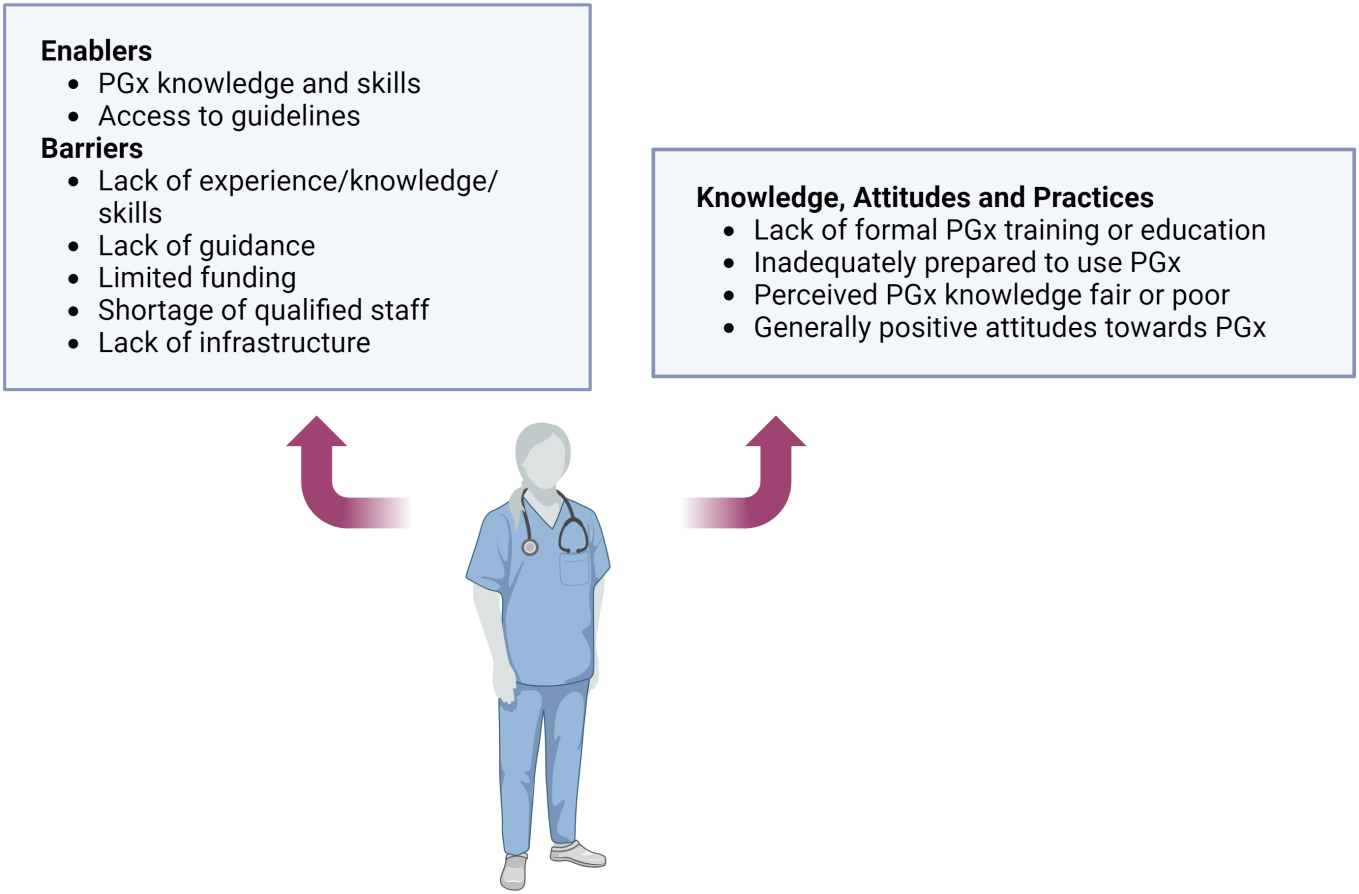
Key themes and attributes identified within healthcare professional surveys.^17^, ^18^, ^19^ Created with BioRender.com. PGx, pharmacogenomics.

KAP related to pharmacogenomics from a healthcare professional perspective arising from this review are summarized in Table 4 with barrier and enabler themes annotated in Figure 3. In all three studies, the majority of survey respondents had not received pharmacogenomic training or education.^17^, ^18^, ^19^ In Dodson's study, most participants rated their knowledge of pharmacogenomics as fair or poor, with prior experience, oncology qualifications, and previous genomic education found to be significant indicators of pharmacogenomic knowledge.^17^

**TABLE 4:** Attributes of healthcare professional knowledge, attitudes, and practice related to pharmacogenomics in pediatric oncology of included studies.^17^, ^18^, ^19^

	Attribute	Number of studies (*n*)
Knowledge	1. Majority of respondents rated perceived knowledge of PGx as fair or poor^17^	1
	2. Majority had not received PGx training or education^17^, ^18^, ^19^	3
	3. Oncology certification, previous genomic education and prior experience were indicators of actual PGx knowledge^17^	1
Attitudes	1. Respondents felt inadequately prepared to order or use PGx testing^18^	1
	2. Overall attitudes to PGx testing were favorable^17^, ^19^	2
	3. Attitudes to PGx testing in oncology were favorable^17^	1
	4. More positive attitudes to PGx testing were affected by communication behavior, observability, prior experience, perceived need and relative advantage^17^	1
	5. Vast majority of respondents perceived PGx implementation would decrease ADRs^17^	1
	6. More negative attitudes were observed in healthcare professionals who used fewer information sources, had less PGx experience, felt PGx was not part of their practice and who had lower perceived need for PGx testing^17^	1
	7. Higher comfort level using PGx testing with improved access to guidelines or interpretation services^18^	1
	8. Increased comfort with a genetics professional or pharmacist collaboration^18^	1
	9. Pharmacists were more interested than physicians in future PGx training and education^19^	1
	10. Few healthcare professionals considered counseling patients on PGx test results their responsibility^19^	1
	11. Most agreed genetic information should be available in the electronic medical record^19^	1
Practice	1. Less than half the respondents had cared for a patient who had received a PGx test (to their knowledge)^17^	1
	2. Most had never educated a patient regarding PGx testing or advocated for a patient to undergo testing^17^	1
	3. Almost half the respondents were unsure whether PGx tests were available at their institution^17^	1
	4. PGx testing outside protocol mandated thiopurine methyltransferase (TPMT) testing was rare^18^	1

Abbreviations: ADR, adverse drug reaction; PGx, pharmacogenomics.

Two studies reported favorable overall attitudes of healthcare professionals with respect to pharmacogenomic testing.^17^, ^19^ One participant group was specifically asked in relation to oncology.^17^ Indicators of negative and positive attitudes to pharmacogenomics were identified by Dodson who found a positive attitude was affected by prior experience of, perceived need, and relative advantage of pharmacogenomic testing.^17^ Conversely, more negative attitudes were observed in healthcare professionals who used fewer information sources in their practice, had less pharmacogenomics experience and had a lower perceived need for pharmacogenomic testing.^17^ The vast majority of participants perceived that pharmacogenomics implementation would decrease ADRs.^17^ Furthermore, healthcare professionals felt inadequately prepared to order or use pharmacogenomic testing,^18^ but indicated that their comfort with using testing would increase with improved access to guidelines, results interpretation services, or collaboration with a field expert.^18^

Healthcare professionals agreed genetic information should be available in a patient's electronic medical record, but few considered counseling patients on pharmacogenomic results to be within their scope of practice.^19^ The healthcare professional groups in two of the studies acknowledged limited experience with pharmacogenomic testing with prescribers rarely ordering tests outside those required in treatment protocols^18^ and a minority of nurses having cared for a patient who had undergone testing.^17^

### 3.6 Key enablers and barriers to pharmacogenomics

One of the key outcomes of the systematic review was to identify enablers and barriers to the implementation of pharmacogenomics (summarized in Table 5). All three studies identified education as an enabler for widespread pharmacogenomics implementation.^17^, ^18^, ^19^ This emerged in both healthcare professional and caregiver groups. From a healthcare professional perspective, improved access to pharmacogenomic guidelines and multi‐disciplinary expert collaboration were also considered enablers in one study.^18^

**TABLE 5:** Enablers and barriers to pharmacogenomics implementation in pediatric oncology as identified in included studies.^17^, ^18^, ^19^

	Theme	Method Professional survey (*n*; studies identifying)	Consumer survey (*n*; studies identifying)
Enablers	1. Education	3	1
	2. Access to guidelines	1	
	3. Multidisciplinary collaboration	1	
Barriers	1. Health/life insurance discrimination		1
	2. Cost of test	1	1
	3. Electronic data sharing/privacy		1
	4. Lack of education/skills	3	
	5. Lack of guidelines	2	
	6. Limited funding	1	
	7. Shortage of qualified personnel	1	
	8. Lack of testing infrastructure	1	

Overall, there were more barriers identified than enablers. In addition to the lack of education, skills, and guidelines that were described in all healthcare professional groups, one study reported limited funding, shortage of qualified staff, and lack of infrastructure as barriers.^19^ Barriers identified from consumer surveys were the possibility of health or life insurance discrimination, the cost of testing to the consumer, data sharing, and privacy concerns.^18^

### 3.7 Approaches to pharmacogenomic education

None of the studies described an educational approach to pharmacogenomics implementation for pediatric patients with oncology.

## 4 DISCUSSION

This review aimed to better understand barriers and enablers to pediatric oncology pharmacogenomics implementation by examining the literature for KAP outcomes and education approaches. While the field of pharmacogenomic research has been established for many decades, widespread implementation has not yet occurred.^1^ Hence, unsurprisingly, both consumer and healthcare professional groups report a lack of experience with pharmacogenomic testing.^17^, ^18^, ^19^ Lack of experience in genetic testing has previously been identified as a barrier to rapid whole genome testing within general pediatrics by consumers and healthcare professionals.^20^ A national Australian study implementing rapid genome testing in critically unwell children found the clinical impact of practitioners having limited experience or awareness of genetics referral pathways resulted in referral delays for testing.^20^ Similarly, a pediatric study of non‐syndromic genetic testing in patients with an Autism diagnosis, surveying healthcare professionals, found more than 50% of respondents felt uncomfortable with the process for genetic testing authorization.^21^ Lack of experience of healthcare professionals has been cited as a major challenge broadly across multiple sub‐specialized fields of genetics implementation.^22^

Despite the reported lack of experience among healthcare professionals and consumers, it was encouraging that those surveyed reported positive attitudes toward pharmacogenomic testing.^17^, ^19^ This finding reflects parents' attitudes toward childhood genetic testing more broadly, in a review representing the views of 3934 parents.^23^ Parents have reported positive attitudes to genetic testing for children across a range of different genetic tests of differing clinical utility.^23^ A positive attitude of healthcare professionals toward rapid whole‐exome sequencing in acute care emerged as a major theme in a survey of 307 healthcare professionals across eight institutes in the United States.^24^ Interestingly, the degree of positivity was strongly influenced by perceived knowledge.^24^ In our systematic review, the positive attitudes of healthcare professionals were driven by a perceived clinical need for, and advantages of, pharmacogenomics testing.^17^, ^18^ Healthcare professionals have also demonstrated positive attitudes to genetic testing across a range of clinical indications including cancer predisposition syndromes,^25^ disability,^26^ and acute critical illness.^20^

While healthcare professionals and consumers were positive about pharmacogenomics testing, their self‐identified lack of experience speaks to the importance of education and training in this field. Indeed, both patients and healthcare professionals are crucial stakeholders for targeted education when introducing genomic testing into clinical practice.^22^ Our review aimed to include educational approaches to pediatric pharmacogenomic oncology programs, however, none of the articles described an educational approach or an assessment of one. The collective focus of the studies included was KAP outcomes. While Mowbray et al.^18^ administered a second questionnaire to health professionals 15 months after the first, showing improvement in pharmacogenomic acceptability, any education provided in the intervening period was not detailed. It is surprising that an approach to the education of pediatric oncology healthcare professionals, patients, or their carers has not been reported or evaluated, despite the existence of actionable pharmacogenomic variant guidelines for cancer and supportive care medications.^5^, ^27^ Successful educational strategies are critical to determining the success of a pharmacogenomics program,^1^, ^28^ and education initiatives should be embedded in every step of implementation. Key education topics that have been outlined for the integration of genomic testing into pediatric practice broadly include background information (basic genetic concepts, overview of genomic testing in children); specific testing relevant to clinical genomic testing (differentiate current test from previous tests ordered, who will be tested, turn‐around time, type of results available, potential for uncertainty, whose results will be reported); psychosocial implications (personal, nuclear and extended family members; cascade testing; insurance implications), and post‐test issues (sample storage, data storage, research opportunities).^22^, ^29^ This framework could be used to scaffold an education approach for pediatric oncology pharmacogenomics implementation. Additional topics that may need inclusion in the pharmacogenomics context include the difference between cancer genetics and pharmacogenomics and the advantages of pharmacogenomics implementation during cancer therapy to reduce ADRs.

One important gap identified in the literature was the absence of opinions or experiences of adolescent and young adult (AYA) patients. None of the identified studies reported on KAP outcomes from a patient perspective in this important cohort. The single consumer group surveyed in the Mowbray et al. study was composed entirely of caregivers.^18^ This is particularly pertinent when we consider that genetic results are relevant for the duration of a person's life, thus caregiver decisions surrounding genetic testing may have prolonged ramifications.^30^ Recent best practice statements for clinical genetic testing specify the need to engage with adolescents and elicit, consider, and respect an adolescent's concerns.^31^ A study by the American College of Genetics and Genomics found that 98% of adolescents want to be involved in the decision‐making with respect to their genetic test results and 53% want to make choices about their own genetic information independently.^31^ Of interest, the actionability of genetic test results was a common driver for adolescents’ desire to learn more about their genetic results in addition to a preference for knowledge about their own health.^31^ These themes are very relevant to pharmacogenomic testing where approximately 80% of pediatric patients will have a high‐risk (and potentially actionable) genotype,^32^ and results impact medication prescribing across a lifetime.

All three studies examined identified education as an enabler of pediatric pharmacogenomics implementation for both healthcare professionals and consumers.^17^, ^18^, ^19^ Further enablers specific to healthcare professionals included expert guidance and collaboration.^18^ These determinants have also previously been acknowledged in the literature,^28^ with respect to pediatric pharmacogenomics implementation. Large collaborative efforts internationally have resulted in freely available, evidence‐based, expert peer‐reviewed, and updated pharmacogenetic clinical practice guidelines consortiums.^5^, ^27^ Collaboration in pediatric pharmacogenomics is essential to advancing knowledge in this specialist field allowing children to benefit from shared implementation lessons, both locally and internationally. However, guidance and collaboration should be used to enhance existing evidence‐based guidelines, not in place of.

Despite collaborative evidence‐based guideline availability and accessibility, implementation in pediatric oncology has been limited. To date, TPMT, glucose‐6‐phosphate dehydrogenase (G6PD), and Nudix Hydrolase 15 (NUDT15) are the only genes screened routinely within pediatric oncology collaborative groups.^33^, ^34^ Key implementation barriers include limited funding, infrastructure, and shortage of qualified staff,^19^ and are well documented in both adult and pediatric cohorts.^1^ However, advances over the last 5 years internationally should soon start to mitigate these barriers. Genomic testing is becoming more widespread, driving down costs and improving accessibility.^28^ Pharmacogenomic testing infrastructure is improving and with it the capacity to integrate results into the electronic medical record.^1^, ^19^, ^28^ As evidence for the clinical utility and cost‐effectiveness of pre‐emptive pharmacogenomic testing in pediatrics builds, there will be an incentive for improved funding pathways and additional infrastructure. Additional funding and education will, in turn, help overcome the shortage of qualified staff and future‐proof workforce development and retention.

The consumer identified barriers to pharmacogenomics implementation differed considerably from those identified by healthcare professionals. Consumers identified the insurance implications of returning an abnormal genetic result as of significant importance to them. The results of many previous studies highlight patients’ or parents' concerns about becoming the victim of genetic discrimination, particularly as it relates to insurance. In a study spanning the United States, Canada, and Australia, interviewing potential carriers of Huntington's Disease, 77% of respondents were concerned about insurance discrimination.^35^ These findings have been mirrored by parents whose children are undergoing screening for autosomal dominant or monogenetic disorders.^36^, ^37^, ^38^ Legal protection exists in law to prohibit discrimination on the basis of genetic information with respect to health insurance and employment (e.g. The Genetic Information Non‐Discrimination Act 2008 (United States),^39^ The Equality Act 2010 (United Kingdom),^40^ and Disability Discrimination Act 1992(Cth) (AU)^41^, ^42^) however this legislation is not exhaustive, nor does it specifically address the full expanse of insurance cover (i.e. life insurance, long‐term care or disability insurance). Furthermore, adherence to the legislation is of concern. A recent Australian study of 174 consumers with cancer predisposition variants found that 77% of those surveyed experienced difficulties related to life insurance.^42^ Of those experiencing difficulties with attaining insurance coverage, 50% had no prior history or symptoms of cancer and had undertaken risk reduction through surveillance or preventative surgery.^42^ Broadly, there is a desire to update both the legislation and industry self‐regulation to reflect a complete ban on the use of genetic test results in insurance underwriting. These findings are relevant when considering national and international pharmacogenomics implementation and the need to engage government stakeholders to increase adherence to current protective legislation.

In our review, consumers were also concerned about data sharing, privacy, and paying for genetic testing.^18^ These barriers have also been discussed broadly in the context of genetic testing in both children and adults from a consumer perspective.^21^, ^23^, ^43^ Efforts must be made to ensure health equity across socio‐economic groups when implementing pharmacogenomics so that patients aren't disadvantaged by prohibitive costs. This will require co‐operation between insurance companies, institutions, and the government. The issue of payment may be less of a concern in countries where universal public healthcare includes genetic testing, such as France, Spain, and Israel.^21^ However, genetic testing for autism spectrum disorders, as an example, remains underutilized in these countries despite low‐patient costs,^21^ indicating that patient payment is not the sole barrier to implementation. Concerns regarding data sharing and privacy can be allayed through robust policy development, legal frameworks, and the provision of patient education. In overcoming these barriers, in addition to testing costs, consumers also need to understand and recognize the advantages that pharmacogene testing and personalized prescribing provide. Lim et al. reviewed parents' attitudes toward genetic testing of children and found that parents believe the advantages outweigh the disadvantages of genetic testing where there is proven clinical benefit to the child.^23^

### 4.1 Limitations

Very few studies fulfilled this review's inclusion criteria. The small number meant results were heterogeneous. We limited the search to those published in English, which may have excluded additional studies. Two of the studies included were conducted in the United States, potentially biasing results to reflect this specific healthcare system. While the studies included were judged to be good quality overall, two of the articles were not clear on how healthcare professionals were selected from a potential pool, or what proportion of the total was involved.^18^, ^19^ One of the articles was included because there was at least one participant that held a pediatric oncology qualification, but it was unclear how many others were experts in pediatric healthcare.^17^ Despite reporting a greater response rate than previous similar surveys, one included article still had a low response rate (14%).^17^ This review also assumed pediatric centers included AYA patients but this was not specifically annotated.

### 4.2 Future directions

While this review has revealed a paucity of literature with respect to the barriers and enablers for pharmacogenomics implementation in pediatric oncology, it provides opportunities and frameworks for improving the field. Future directions include definitively answering critical research questions including proving ADR reduction and health economic benefits of pre‐emptive pharmacogenomic testing in pediatric oncology practice.^44^ In addition, implementation science can be used to craft healthcare professional and consumer education packages to overcome highlighted barriers in both the adult and pediatric sectors.

## 5 CONCLUSIONS

This review has highlighted a lack of studies describing knowledge, attitudes, practices, and educational approaches for pharmacogenomics within pediatric oncology. Despite the limited number of studies identified, the concerns of both healthcare professionals and consumers are similar to those raised in pediatric genetics literature more broadly. This review highlights some specific areas of importance to be considered when trying to overcome the described barriers and enhance enablers of pharmacogenomics implementation. First, ensuring all stakeholders have been involved in discussions will be important, including widespread engagement with AYA patients. Targeted education programs for both consumers and healthcare professionals are critical to the success of implementation. Considered national approaches to funding models for pharmacogenomic testing will be imperative to enable more rapid uptake and reimbursement. Finally, eliciting local government assistance in ensuring adherence to existing laws and preventing the use of genetic test results in insurance underwriting will reassure parents and patients of their protection when participating in pharmacogenomic testing. We have demonstrated the need for further research to ascertain consumer and healthcare professional perspectives on pharmacogenomics in pediatric oncology, and in turn, identify implementation enablers and barriers.

## AUTHORS CONTRIBUTIONS

I can confirm all authors contributed to manuscript writing.

## FUNDING INFORMATION

This study was funded by the Kids Cancer Project MARVEL‐PIC Grant together with the Victorian Pediatric Cancer Consortium. The funder of the study had no role in the study design, data collection, data analysis, data interpretation, or the writing of this report.

## DISCLOSURES

All the authors declare no conflict of interest.

## ETHICS STATEMENT

This study is approved under institute Human Ethics and Research Committee through Murdoch Children’s Research Institute [HREC 89083]. The contributing authors all meet criteria as defined within the author guidelines. CM lead the systematic review and conceptualisation of the manuscript. All other authors contributed in the process of article curation [TS, CM, RC], initial search criteria and methodology writing [SL] or editing [all authors]. Full length of curated articles can be accessed by contacting CM or RC directly.

## Supporting information


Appendix S1.

## Data Availability

Data is available upon request.
