# Supplementary material for: A systematic review of knowledge, attitude and practice of pharmacogenomics in pediatric oncology patients
Source: Pharmacol Res Perspect. 2023 Nov 27;11(6):e01150. doi: 10.1002/prp2.1150 (PMC10682497; doi:10.1002/prp2.1150)
Supplement: Supplementary file 1 — Appendix S1. [file PRP2-11-e01150-s001.docx]

Database: Ovid MEDLINE(R) ALL <1946 to November 14, 2022>

Search Strategy:

--------------------------------------------------------------------------------

1     exp pharmacogenetics/ or exp pharmacogenomic testing/ (14038)

2     (pharmacogenetic* or pharmacogenomic* or pgx or capgx).tw,kf. (20309)

3     1 or 2 (25135)

4     exp neoplasms/ or exp stem cell transplantation/ or exp bone marrow transplantation/ or exp allografts/ (3854529)

5     (cancer* or carcinoma* or neoplas* or tumo?r* or malignan* or oncolog* or metasta* or leuk?emia* or lymphoma*).tw,kf. (4206318)

6     (((stem cell or bone marrow or h?ematopoietic or allogeneic or autologous or homologous or allograft or autograft) adj3 transplant*) or (bmt or sct)).tw,kf. (127696)

7     4 or 5 or 6 (5137420)

8     exp Practice Patterns, Physicians'/ or exp Health Knowledge, Attitudes, Practice/ or exp "Attitude of Health Personnel"/ (334495)

9     ((physician* or clinician* or clinical staff or clinical practice or pharmacist* or pharmacy or pharmacies or oncologist* or oncology provider* or doctor* or nurse* or health professional* or health care professional* or healthcare professional* or healthcare worker* or health care worker* or healthcare provider* or health care provider* or healthcare personnel or health care personnel or health personnel or medical staff or medical practitioner* or general practitioner* or genetic counsellor* or care-giver* or caregiver* or carer* or patient* or consumer* or participant*) adj7 (knowledge* or understanding or misunderstanding or comprehension or comprehend* or experience or experiences or expertise or receptive* or perspective* or perception* or attitude* or opinion* or accepta* or utili#ation or utility or utili#e or utili#ing or preference* or practice* or education or training or learning or barrier* or difficult* or aware* or engagement or readiness or interest or interests or willingness or unwillingness or implement* or endorse* or support* or participation or embrace* or familiari* or enthusias* or motivat* or confidence)).tw,kf. (1364317)

10     8 or 9 (1578790)

11     exp child/ or exp pediatrics/ or exp infant/ or exp adolescent/ (3919889)

12     (child* or infant* or newborn* or p?ediatric* or juvenile* or adolescen* or teen* or youth).tw,kf. (2495812)

13     11 or 12 (4584837)

14     3 and 7 and 10 and 13 (94)

15     limit 14 to (english language and yr="2012 -Current") (74)

***************************
